# How Accurate Are Home Blood Pressure Devices in Use? A Cross-Sectional Study

**DOI:** 10.1371/journal.pone.0155677

**Published:** 2016-06-01

**Authors:** Marcel Ruzicka, Ayub Akbari, Eva Bruketa, Jeanne Françoise Kayibanda, Claude Baril, Swapnil Hiremath

**Affiliations:** 1 Division of Nephrology, Faculty of Medicine, University of Ottawa, Ottawa, Canada; 2 Division of Cardiology, University of Ottawa Heart Institute, Ottawa, Canada; 3 Division of Nephrology, The Ottawa Hospital, Ottawa, Canada; 4 Clinical Epidemiology Program, Ottawa Hospital Research Institute, Ottawa, Canada; 5 Kidney Research Centre, Ottawa Health Research Institute, Ottawa, Canada; Rouen University Hospital, FRANCE

## Abstract

**Background:**

Out of office blood pressure measurements, using either home monitors or 24 hour ambulatory monitoring, is widely recommended for management of hypertension. Though validation protocols, meant to be used by manufacturers, exist for blood pressure monitors, there is scant data in the literature about the accuracy of home blood pressure monitors in actual clinical practice. We performed a chart review in the blood pressure assessment clinic at a tertiary care centre.

**Methods:**

We assessed the accuracy of home blood pressure monitors used by patients seen in the nephrology clinic in Ottawa between the years 2011 to 2014. We recorded patient demographics and clinical data, including the blood pressure measurements, arm circumference and the manufacturer of the home blood pressure monitor. The average of BP measurements performed with the home blood pressure monitor, were compared to those with the mercury sphygmomanometer. We defined accuracy based on a difference of 5 mm Hg in the blood pressure values between the home monitor and mercury sphygmomanometer readings. The two methods were compared using a Bland-Altman plot and a student’s t-test.

**Results:**

The study included 210 patients. The mean age of the study population was 67 years and 61% was men. The average mid-arm circumference was 32.2 cms. 30% and 32% of the home BP monitors reported a mean systolic and diastolic BP values, respectively, different from the mercury measurements by 5 mm Hg or more. There was no significant difference between the monitors that were accurate versus those that were not when grouped according to the patient characteristics, cuff size or the brand of the home monitor.

**Conclusions:**

An important proportion of home blood pressure monitors used by patients seen in our nephrology clinic were inaccurate. A re-validation of the accuracy and safety of the devices already in use is prudent before relying on these measurements for clinical decisions.

## Introduction

Blood pressure (BP) measurement is the cornerstone for diagnosis and management of hypertension (HTN). Oscillometric BP devices, especially when used by patients at home, offer major advantages over the century old BP assessment by the mercury sphygmomanometer[1]. Firstly, they reduce white coat and masked effect in diagnosis and management of HTN[2]. Secondly, they improve the accuracy of the assessment of overall BP load by BP readings obtained at different times of the day. Finally, they improve patients’ adherence by involving their participation in the HTN management. Not surprisingly, home BP (HBP) monitoring has been reported to be a stronger predictor of cardiovascular morbidity and mortality than office BP measurements[3]. A meta-analysis comparing a strategy of HBP and office BP measurement to guide therapy found that HBP monitoring resulted in a significantly lower BP[4]. Thus many international guidelines recommend the HBP use in HTN management[5–8]. The worldwide reported use of home BP devices varies from 30–70% [9–11].

However, despite the widespread use of HBP monitors, little data exists on the accuracy of the monitors in use. Three standard protocols exist for BP device validation, published by the Association for the Advancement of Medical Instrumentation (AAMI), the British Hypertension Society (BHS) and the European Society of Hypertension respectively[12–14]. The Food and Drug Administration requires that the AAMI standard be used for validation[15] while Hypertension Canada endorses an HBP monitor if validated by any one of the three protocols[5]. However, these protocols are meant for pre-licensing commercial validation, and few reports exist of real world accuracy of HBP monitors. These few studies do report a wide range of inaccuracy (10 to 69%), and are mostly from over a decade ago, from an older generation of monitors[16–18]. In addition, similar to other instruments used in clinical medicine, HBP devices might lose their original safety and accuracy over time. However no recommendation with regards to re-validation process of safety and accuracy of home BP monitors exists.

At our centre, we have been conducting a BP assessment clinic for follow up of hypertensive patients from the nephrology program, with the purpose of providing education about BP management, lifestyle modifications, and to guide self-measurement. Given the importance of home measurement of BP, and the concerns of accuracy, we undertook a chart review with a goal of assessing the accuracy of home BP monitors in our population, and to identify factors that are associated with accuracy of these monitors.

## Material and Methods

A chart review of all patients referred to and seen in the BP assessment clinic from July 1 2011 to Apr 30th 2014 was performed. Only patients who did bring in a home BP monitor to their clinic visit were included in this study. For those who had multiple visits, we only included data from the first visit.

From an electronic database, the chart review involved data abstraction of patient demographics and clinical data, including details of the BP measurements (mercury and home monitor), arm circumference, and also the manufacturer of the HBP monitor. Institutional review board approval, from the Ottawa Health Sciences Research Ethics Board (20140619-01H) was obtained prior to conducting the chart review. Patient records were de-identified prior to analysis. Informed consent was waived given this was a retrospective chart review using de-identified data.

The BP measurements are all taken in a quiet clinic room, after a minimum of 5 minutes of resting. The mercury sphygmomanometer readings are done using a calibrated machine, and by a registered nurse (RN) trained to follow the standardized protocol of the Canadian Coalition for High Blood Pressure Prevention and Control[19]. After measurement of the patients arm circumference, a proper sized cuff is chosen for the mercury measurement. Similarly, the cuff size of the home monitor is checked to be appropriate before proceeding with the measurements. All measurements are taken with the patient sitting upright with a proper back support and with feet uncrossed and placed flat on the floor. The patient’s arm is supported on a table or firm surface. Firstly, on bare arms, mercury readings are taken on both arms to determine if the BP is equal in both arms. The heart rate and the absence of any arrhythmia are confirmed before proceeding. If the BP readings are within 5 mm Hg, the manual cuff is placed on one arm and the home BP cuff on the other arm. Three simultaneous BP measurements are taken by the RN, with one minute wait in between readings, and averaged. If the BP in the two arms varies by more than 5 mm Hg, it is confirmed once again, and once confirmed, 6 sequential measurements are taken in the arm with the higher BP, with three measures using the mercury alternating with three with the home monitor.

In our analysis, we first compared the average of the three measurements with the mercury and the three measurements with the home monitor. A difference of 5 mm Hg in the systolic BP (i.e. either 5 mm Hg or higher, or 5 mm Hg or lower reading with the home monitor compared with mercury) was considered inaccurate[20]. We compared the individual measurements using a Bland-Altman analysis[21]. We also compared average of BP measurement recorded with home BP devices were against the average of BP measurement recorded with mercury device using a student’s t-test. In addition, we performed a comparison of the patient and device characteristics between accurate and inaccurate devices using the chi-square test for nominal variables and the t-test for continuous variables. Lastly, we report inaccuracy also using less stringent criteria of a difference of 10 mm Hg and 20 mm Hg for the systolic BP, and inaccuracy using 5, 10 and 20 mm Hg for the diastolic BP. A p value < 0.05 was considered statistically significant. All analysis was carried out using JMP (version 8.0.1, SAS Inc, Cary NC).

## Results

**Fig 1:**
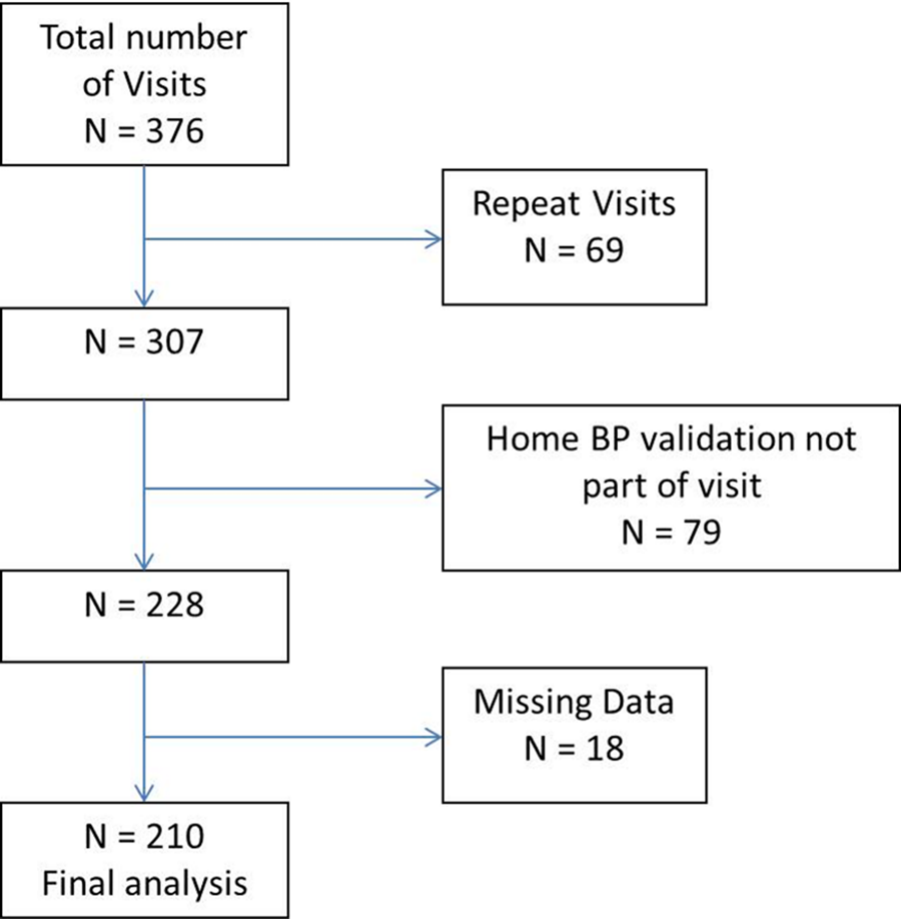
Selection of study population.

376 patient-visits occurred over the study period. 3 patients had home BP devices with mis-match between the cuff size and arm circumference; the nurse did not assess for accuracy of the home BP device in these cases and provided a recommendation to obtain the correct cuff size. These three patients did return with the correct cuff size at a subsequent appointment, and the measurements from that appointment were used in the analysis. After excluding patients who did not bring a home monitor, patients with repeat visits and encounters with missing data, we had data available on 210 patients and home monitors (Fig 1).

About 60% of the study population was men, with a mean age of 67 years (details in Table 1). The average mid-arm circumference was 32.2 cms. The Omron brand (Omron Healthcare, Toronto, Canada) was the most popular, used in just over half the cases. The overall mean systolic as well as diastolic BP measured with mercury was significantly lower than the HBP monitor measurements (p = 0.02).

**Table 1:** Baseline Characteristics of Patients and Home Monitors.

Variables	N = 210
**Patient's characteristics**	
Age (in years)	67 ± 14
Men*	127 (61%)
Body mass index (kg/m^2^)	28.9 ± 6.1
Arm Circumference (in cms)	32.2 ± 5.0
Waist circumference (in cms)	101.2 ± 16.7
Systolic Blood Pressure measurement**	
Mercury sphygmomanometer	132.3 ± 18.5
Home BP monitors	133.4 ± 18.1
Diastolic Blood Pressure measurement**	
Mercury sphygmomanometer	68.9 ± 12.5
Home BP monitors	71.4 ± 11.8
**BP monitors characteristics**	
Monitor Brand*	
Omron	105 (50%)
A&D/Life Source	52 (24.7%)
Others	53 (25.2%)
Cuff Size*	
Small	7 (3%)
Regular	140 (67%)
Large	62 (30%)
Extra-large	1 (0.5%)

All values in mean + standard deviation unless specified

* Percentages are expressed with respect to column totals

**p <0.05 with two-tailed t-test as compared to home monitor

30% (63 of 210) of the home BP monitors reported mean systolic BP values which were 5 mm Hg or more different from the mercury measurements. There was no significant difference between the monitors that were accurate versus those that were not when grouped according to the patient characteristics, cuff size or the brand of the home monitor (Table 2). The absolute difference in the diastolic blood pressure was significantly higher with the devices that were inaccurate for systolic BP than in the accurate devices.

**Table 2:** Patient and BP devices characteristics according to accuracy of systolic BP.

Variables	Accurate* N = 147 (70%)	Inaccurate* N = 63 (30%)	p value
**atient's characteristics**			
Age (in years)	67.0 + 13.4	67.1 + 16.3	0.40
Men (N, %)**	91 (61.9%)	36 (57.1%)	0.11
Body mass index (kg/m^2^)	28.8 ± 6.5	29.1 ± 5.1	0.20
Arm Circumference (in cms)	32.1 ± 4.9	32.4 ± 5.2	0.45
Waist circumference (in cms)	100.8 ± 17.6	102.3 ± 14.3	0.23
Absolute difference in diastolic BP (in mm Hg)	5.4 ± 5.9	3.5 ± 3.4	0.02
**Cuff Size** (N, %)**			
			0.81
Small	4 (2.7%)	3 (4.8%)	
Regular	101 (68.7%)	39 (61.3%)	
Large	42 (28.6%)	20 (32.3%)	
Extra-large		1 (1.6%)	
**Monitor Brand** (N, %)**			
			0.16
Omron	79 (53.7%)	26 (43.3%)	
A&D/Life Source	36 (24.5%)	16 (25.4%)	
Others	32 (21.8%)	21 (33.3%)	

All values in mean ± standard deviation unless specified.

*Accuracy determined by 5 mm Hg difference in SBP

** Percentages are expressed with respect to column totals

For diastolic BP, 67 monitors (32%) reported mean values that were 5 mm Hg or more different than the mercury. Patients with inaccurate devices were more likely to be older men, however there was no significant difference between the other patient characteristics, cuff size or the brand of the home monitor (Table 3). The absolute difference in the systolic blood pressure was significantly higher with the devices that were inaccurate for diastolic BP than in the accurate devices.

**Table 3:** Patient and BP devices characteristics according to accuracy of diastolic BP.

Variables	Accurate* N = 143 (68%)	Inaccurate* N = 67 (32%)	p value
**Patient's characteristics**			
Age (in years)	65.6 ± 14.4	70.0 + 13.8	0.04
Men (N, %)**	95 (66.4%)	32 (47.7%)	0.02
Body mass index (kg/m^2^)	28.5 ± 6.1	29.7 ± 6.1	0.20
Arm Circumference (in cms)	31.9 ± 4.9	32.7 ± 5.3	0.96
Waist circumference (in cms)	101.2 ± 16.7	101.3 ± 16.8	0.29
Absolute difference in diastolic BP (in mm Hg)	3.3 ± 3.3	3.5 ± 3.4	<0.01
**Cuff Size** (N, %)**			0.20
Small	5 (3.5%)	2 (3.0%)	
Regular	101 (70.6%)	39 (58.2%)	
Large	36 (25.2%)	26 (38.8%)	
Extra-large	1 (0.7%)	0	
**Monitor Brand** (N, %)**			0.39
Omron	74 (51.7%)	31 (46.3%)	
A&D/Life Source	37 (25.9%)	15 (22.4%)	
Others	32 (22.4%)	21 (31.3%)	

All values in mean ± standard deviation unless specified.

*Accuracy determined by 5 mm Hg difference in SBP

** Percentages are expressed with respect to column totals

Using a different threshold for accuracy (difference more than 10 mm Hg), 16 of 210 (8%) HBP monitors were inaccurate for systolic BP and 18 (9%) for diastolic BP. Four of 210 HBP monitors (2%) were inaccurate using the 20 mm Hg threshold, for both systolic and diastolic BP. The inter-arm difference in BP was more than 5 mm Hg in 28% of the patients, in whom the accuracy comparison was measured by sequential rather than simultaneous measurements. Sensitivity analysis grouping them by sequential (34% inaccuracy) versus simultaneous (30% inaccuracy) measurement did not find a significant difference.

**Fig 2:**
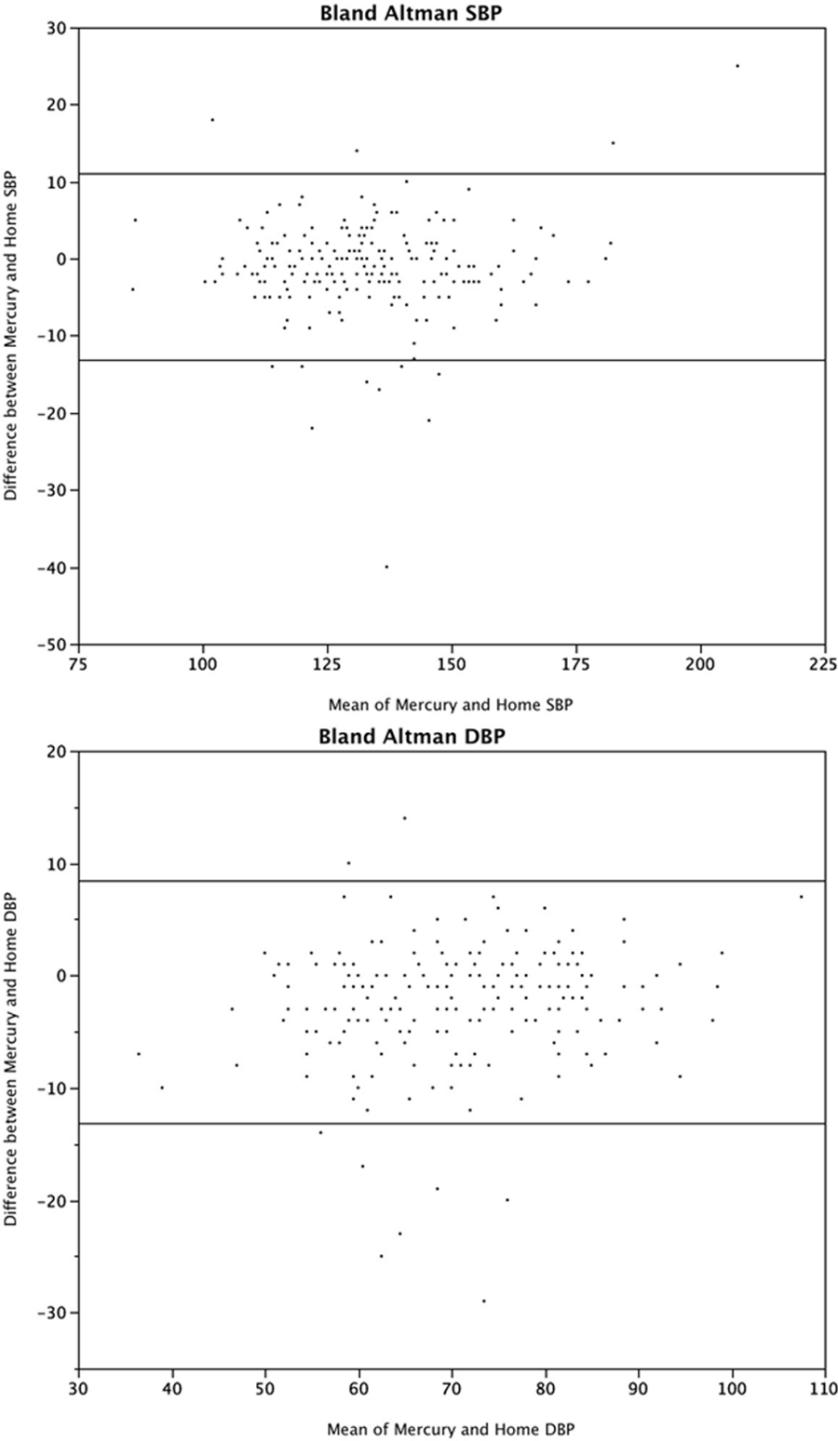
Comparison of systolic and diastolic blood pressure measurements between mercury sphygmomanometer and home blood pressure devices using a Bland Altman plot.

**Fig 3:**
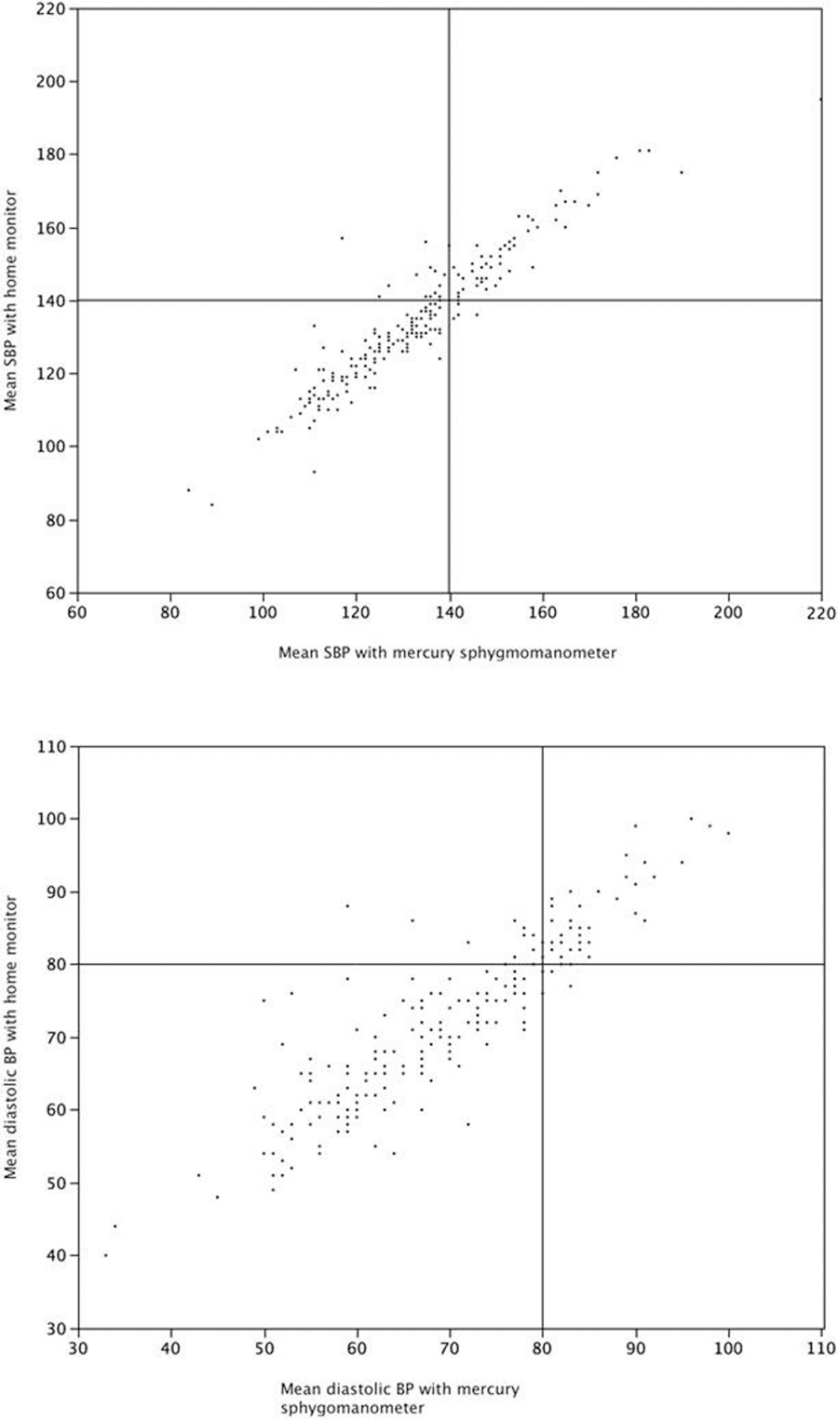
Scatterplots of actual mean systolic and diastolic blood pressures with the two methods. Lines are drawn, for illustration purposes only, through a threshold of 140 mm Hg (for systolic) and 80 mm Hg (for diastolic).

The Bland-Altman analysis is presented in Fig 2. The 95% limits of agreement were -13, +11 mm Hg for systolic BP and -13, +8 mm Hg for diastolic BP. The difference in BP values was spread across the entire range of BP, with no trend of inaccuracy with increasing (or decreasing) blood pressure. Scatterplots to demonstrate the correlation between the blood pressures are presented in Fig 3.

Additional analyses conducted between the inaccurate home BP devices that underestimated SBP (N = 25) by more than 5 mm Hg and those that overestimated SBP (N = 38) by more than 5 mm Hg are presented in Table 4. The patients in whom the home BP device overestimated SBP by more than 5 mm Hg were more likely to be men, with a greater BMI, arm and waist circumference and needing a correspondingly larger cuff compared to those that underestimated SBP. In addition, there was a difference in the device brand, with Omron devices more likely to overestimate BP and A&D/Life source more likely to underestimate BP.

**Table 4:** Patient and BP devices characteristics separated according to over- or underestimation.

Variables	Home monitor underestimated SBP* (N = 25)	Home monitor overestimated SBP* (N = 38)	p value
**Patient's characteristics**			
Age (in years)	67.0 ± 14.9	67.2 ± 17.5	0.97
Men (N, %)**	10 (40%)	26 (68.4%)	0.03
Body mass index (kg/m^2^)	26.1 ± 5.1	30.9 ± 4.2	0.03
Arm Circumference (in cms)	30.9 ± 7.2	33.4 ± 3.3	0.06
Waist circumference (in cms)	93.4 ± 12.5	107.8 ± 12.5	< 0.01
**Cuff Size** (N, %)**			
			0.02
Small	3	0	
Regular	17	22	
Large	4	16	
Extra-large	1	0	
**Monitor Brand** (N, %)**			
			<0.01
Omron	8	18	
A&D/Life Source	13	3	
Others	4	17	

All values in mean ± standard deviation unless specified.

*Under or over-estimation determined by 5 mm Hg difference in SBP

** Percentages are expressed with respect to column totals

## Discussion

In this cross-sectional study with 210 patients and HBP monitors, we report a relatively high proportion of inaccurate HBP monitors. Though in the majority of the HBP monitors, the difference between readings by auscultation technique using mercury sphygmomanometer and by the automated oscillometric HBP device was small (<5 mmHg), in a disturbingly high proportion, the differences were greater than 5 mm Hg. These large differences between the measurements could have an adverse impact on the diagnosis and management of HTN. We therefore believe that we have identified a significant gap in the HBP monitoring process which needs to be further investigated, and if confirmed, addressed.

Similar to our findings, previous studies have also showed a significant proportion of inaccurate home BP devices [16–18, 22–28]. However, differences in methodologies used in these past studies including the generation of HBP monitors assessed, threshold used to determine inaccuracy, type and number of observers who assessed the accuracy limit the comparison of our results to those reported by these studies. For example, in a Canadian study published in 2001, Campbell et al. observed that 35% of home BP devices were inaccurate based on a threshold difference of 4 mmHg or more between BP values recorded by home BP devices and those indicated by mercury sphygmomanometer [25]. In USA, Merrick et al reported that 34% of home BP monitors have recorded BP values out of a threshold difference of 10mmHg in comparison with BP values indicated by a mercury sphygmomanometer. In this study published in 1997, the comparison was between measurements taken by the patient compared to those by a technician, with a significant proportion of aneroid machines included in the mix [17]. In 1984, Hahn et al report an 11% inaccuracy proportion using a 7 mm Hg threshold, however, again with mostly aneroid monitors (only 4% digital monitors)[16]. Studies from other countries have also reported a higher proportion of home BP monitors that were inaccurate. In Turkey, Dilek et al showed that 59.3% of home BP monitors were inaccurate [27] and Wong et al in China observed that errors for systolic and diastolic BP readings occurred in 62% and 64% of home BP monitors respectively[28].

Our study has several major strengths compared to the previous data. The evaluation process took place in the tertiary care hospital-based HTN clinic attended by a nurse specialized and trained in the management of patients with HTN, thus minimizing human error in BP measurement. Secondly, our evaluation process included repeated simultaneous measurements of BP by mercury sphygmomanometer and HBP monitor. In addition, we identified that there are differences in patient (more men with higher BMI) and device (brand) characteristics which overestimate SBP in comparison to those that underestimate SBP. Lastly, the results did not vary meaningfully with sensitivity analyses, and were consistent using different thresholds.

Our study does have also certain limitations. Firstly, we have information on the brand of the HBP, but not series or model numbers to determine their validation status. In addition, we were unable to track the age of the each individual device. Thus we cannot comment on whether and to what extent the age of the device plays a role in the accuracy of measurements. Secondly, our study was designed as retrospective analysis of collected data, and as much as the results are provocative, they should be interpreted with caution. Third, as the measurements were done in routine clinical practice, only one RN measured the BP. However, the agreement between the home BP monitor and the manual measurements may have been exaggerated given that the RN was not blinded to the previous device readings, thus any bias, if present, would be towards the null, thus making our findings of inaccuracy more robust. The differences that we report between devices that overestimate SBP and those that underestimate SBP are subject to multiple comparison fallacy, and hence should be considered hypothesis generating, though worthy of more studies. Lastly, it is possible that there could be selection bias in the overall study, since the study could only include data on patients who did monitor BP at home and were willing to bring it in for the clinic visit.

In conclusion, we believe that we have identified a significant gap in the HBP monitoring process which needs to be further investigated, and if confirmed, addressed. In fact our study raises the issues relating to the re-validation of the accuracy and safety of the devices already in use. Currently standard international protocols developed by the Association for the Advancement of Medical Instrumentation (AAMI) [29], the British Hypertension Society (BHS) [13] and the European Society of Hypertension [30] exist for pre-licensing commercial validation of BP devices. The validation methods proposed by these protocols are difficult to implement in a routine clinical care especially because they are time consuming and require more technicians to perform. Thus, there is a lack of a process of re-validation of the accuracy and safety of the devices already in use. This leaves the patient and the health care practitioner with unanswered questions of where and how frequently HBP device needs to be re-validated. In the worst case, the treatment decision on HTN management would be based on possibly inaccurate measurements from home BP devices.

## Conclusions

Home BP monitoring can improve the accuracy of HTN diagnosis, and be quite valuable for HTN management. However, the overall benefit from HBP monitoring can be offset by inaccuracy of the device itself. The magnitude of this inaccuracy in specific cases could indeed lead to an adverse impact in management of HTN. Thus, increased awareness and detection of this phenomenon is warranted.
